# Comprehensive Genomic Analysis Reveals the Prognostic Role of *LRRK2* Copy-Number Variations in Human Malignancies

**DOI:** 10.3390/genes11080846

**Published:** 2020-07-24

**Authors:** Gianluca Lopez, Giulia Lazzeri, Alessandra Rappa, Giuseppe Isimbaldi, Fulvia Milena Cribiù, Elena Guerini-Rocco, Stefano Ferrero, Valentina Vaira, Alessio Di Fonzo

**Affiliations:** 1 Pathology Unit, Fondazione IRCCS Ca’ Granda–Ospedale Maggiore Policlinico, 20122 Milan, Italy; fulviamilena.cribiu@policlinico.mi.it (F.M.C.); stefano.ferrero@unimi.it (S.F.); valentina.vaira@unimi.it (V.V.); 2 School of Pathology, University of Milan, 20122 Milan, Italy; 3 Neurology Unit, Fondazione IRCCS Ca’ Granda–Ospedale Maggiore Policlinico, 20122 Milan, Italy; giulia.lazzeri@unimi.it (G.L.); alessio.difonzo@policlinico.mi.it (A.D.F.); 4 Dino Ferrari Center, Neuroscience Section, Department of Pathophysiology and Transplantation, University of Milan, 20122 Milan, Italy; 5 School of Neurology, University of Milan, 20122 Milan, Italy; 6 European Institute of Oncology (IEO), 20141 Milan, Italy; alessandra.rappa@ieo.it (A.R.); elena.guerini@unimi.it (E.G.-R.); 7 Unit of Surgical Pathology and Cytogenetics, ASST Grande Ospedale Metropolitano Niguarda, 20162 Milan, Italy; giuseppe.ismbaldi@ospedaleniguarda.it; 8 Department of Oncology and Hemato-oncology, University of Milan, 20122 Milan, Italy; 9 Department of Biomedical, Surgical, and Dental Sciences, University of Milan, 20122 Milan, Italy

**Keywords:** LRRK2, cancer, mutations, CNV, prognostic

## Abstract

Genetic alterations of leucine-rich repeat kinase 2 (*LRRK2*), one of the most important contributors to familial Parkinson’s disease (PD), have been hypothesized to play a role in cancer development due to demographical and preclinical data. Here, we sought to define the prevalence and prognostic significance of *LRRK2* somatic mutations across all types of human malignancies by querying the publicly available online genomic database cBioPortal. Ninety-six different studies with 14,041 cases were included in the analysis, and 761/14,041 (5.4%) showed genetic alterations in *LRRK2*. Among these, 585 (76.9%) were point mutations, indels or fusions, 168 (22.1%) were copy number variations (CNVs), and 8 (1.0%) showed both types of alterations. One case showed the somatic mutation R1441C. A significant difference in terms of overall survival (OS) was noted between cases harboring somatic *LRRK2* whole deletions, amplifications, and CNV-unaltered cases (median OS: 20.09, 57.40, and 106.57 months, respectively; *p* = 0.0008). These results suggest that both *LRRK2* amplifications and whole gene deletions could play a role in cancer development, paving the way for future research in terms of potential treatment with LRRK2 small molecule inhibitors for *LRRK2*-amplified cases.

## 1. Introduction

Identification of the PARK8 locus [1] and mutations in the leucine-rich repeat kinase 2 (*LRRK2*) gene [2,3], located on chromosome 12 (12q12) in familial cases of Parkinson’s disease (PD), more than fifteen years ago is considered a game-changing discovery in our knowledge of this yet incurable neurodegenerative disorder. Today, single *LRRK2* mutations represent one of the most frequently known genetic determinants of PD [4].

The *LRRK2* gene consists of 51 exons, and it encodes a large protein of 2527 amino acids, retaining two catalytic domains with kinase (MAPKKK domain) and GTPase (ROC, Ras in Complex domain) function and other protein–protein interaction domains (armadillo-like, leucine-rich repeats, WD40) [5]. All PD-associated mutations identified until now are single nucleotide substitutions, the most relevant being G2019S, which accounts for approximately half of *LRRK2* mutation in Caucasian populations, underlying approximately 5% of PD cases of autosomal dominant PD and approximately 2% of PD cases with no known family history [6,7,8], and R1441C/G/H. Interestingly, these mutations cluster in the two aforementioned enzymatic domains. The gene encodes for the LRRK2 protein, also known as dardarin, which is widely expressed in different tissues, namely the brain, heart, kidney, and lungs [9], but also in peripheral blood mononuclear cells (PBMCs), including lymphocytes and monocytes [10].

The physiological role of LRRK2 is still not completely understood, despite the enormous amount of research conducted on this protein in the last years. LRRK2 has been hypothesized to have a role in several fundamental cellular processes, primarily autophagy, endocytosis, and mitochondrial and cytoskeletal function [11,12,13,14]. Notably, disruptions in all these processes have been implicated in the neurodegeneration leading to PD [15,16,17,18]. Furthermore, as predictable from the expression of the protein in PBMCs, LRRK2 alterations have also been implicated in dysfunction of immune pathways [19]. Interestingly, genome-wide association studies have linked *LRRK2* to at least three chronic inflammatory conditions, namely Crohn’s Disease [20,21,22], leprosy [23], and tuberculosis [24]. Taken together, current evidence strongly suggests a role of LRRK2 misfunction in the pathogenesis of PD, possibly mediated by a role in neuroinflammation [25].

Current evidence in the literature may suggest a negative association between certain neurological diseases like PD or Alzheimer’s disease and cancer [26]. Interestingly, demographical data suggest an increased incidence of cancer in individuals with a germline G2019S *LRRK2* mutation, in particular, melanoma [27,28,29,30]; however, findings are not consistent across all studies [31,32]. Moreover, in vitro models recently demonstrated that downregulation of LRRK2 suppresses cholangiocarcinoma cell growth [33] and decreases proliferation of papillary thyroid carcinoma [34]; on the other hand, overexpression of LRRK2 activates survival and proliferation signals in melanocytes and melanoma cells [35]. A series of molecular studies have linked the abnormal activity of this protein to autophagic pathways, inflammation, and mitochondrial dysfunction [11,12,13,14,19], but a defined role in cancer pathogenesis needs yet to be elucidated. Several mechanisms for which LRRK2 could display a tumor-suppressor function have been described, most notably p53 phosphorylation and p21 induction [36], JNK activation [37], and RCAN1 phosphorylation [38]. In contrast, LRRK2 has also a potentially oncogenic role in MET signaling activation [39]. Using multigene testing panels, *LRRK2* demonstrated a prognostic significance in different types of cancers, including oral squamous cell carcinomas [40], intrahepatic cholangiocarcinoma [33], non-small-cell lung cancer [41,42], and colon cancer [43]. Recently, great efforts to develop small molecule inhibitors of LRRK2 have been made, hoping for a disease-modifying role in the setting of PD [44,45,46,47]. If an oncogenic role of LRRK2 is confirmed, those drugs are of potential use in the field of cancer-targeted therapy as well.

The current study aims to establish the prevalence of *LRRK2* point mutations, indels or fusions (hereby MUTs) and copy-number variations (CNVs) in a large cohort of human malignancies, as well to investigate the potential prognostic value of such genetic alterations.

## 2. Materials and Methods

A curated set of 176 non-redundant studies publicly available in the online cancer genomic database cBioPortal [48,49,50], comprising 46,595 samples from all types of human malignancies, was initially screened, assessing whether *LRRK2* was profiled across those publications; 96 studies endured the screening (full list available in Table S1), encompassing 23,796 different samples from all human cancers excluding those of the eye, the peripheral nervous system, and the thymus. Cases without both MUTs and CNVs data were excluded; a total of 14,286 samples from 14,041 patients remained. The dataset was also queried for MUTs alone, comprising 17,454 patients from 94 studies (2 studies excluded for not profiling *LRRK2* MUTs), and for CNVs alone, comprising 15,183 patients from 93 studies (3 studies excluded for not profiling *LRRK2* CNVs). For survival analysis of the different types of CNVs, (i.e., amplifications and whole gene deletions), the raw data were downloaded from the cBioPortal database and processed with MedCalc Statistical Software version 19.1.3 (MedCalc Software, Ostend, Belgium). Overall survival was calculated using the Kaplan–Meier Estimate; statistical significance was calculated with a Log-rank Test. Statistical significance of co-occurrence of alterations of LRRK2 and other genes was calculated with Fisher Exact test for *p*-values and with the Benjamini–Hochberg procedure for *q*-values.

## 3. Results

**Figure 1:**
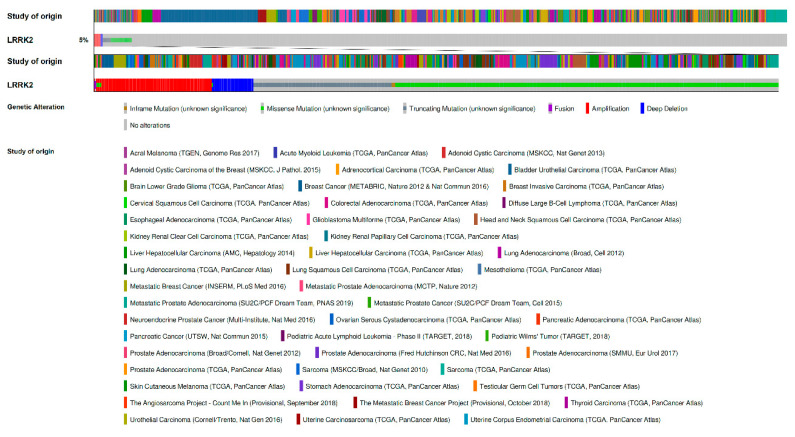
Prevalence and distribution of *LRRK2* genetic alterations across the analyzed studies. The 5% of the first and second row is expanded to better visualize the data [48,49,50].

Among 96 non-redundant studies publicly available in the online cancer genomic database cBioPortal [48,49,50], comprising 46,595 samples from all types of human malignancies, the overall observed prevalence of *LRRK2* genetic alterations was 761/14041 (5.4%), with MUTs accounting for 585 cases (76.9%; 426 missense, 155 truncating, 3 inframe indels, 1 fusion) and CNVs accounting for 168 cases (22.1%; 123 amplifications and 45 whole gene deletions); eight cases (1.0%) showed both MUTs and CNVs (5 missense and amplification, 1 fusion and amplification, 1 missense and whole gene deletion, 1 inframe indel and amplification), as shown in Figure 1. All these were somatic alterations, and no germinal *LRRK2* mutations were present in the dataset.

**Figure 2:**
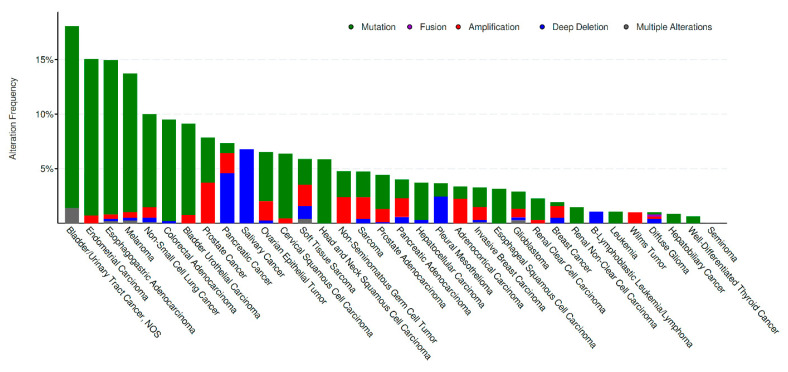
*LRRK2* alteration frequency across cancer types. The types of alterations are color-coded as shown in the legend above [48,49,50].

When considering studies with at least 50 patients profiled, alterations across different cancer types ranged from 18.06% (bladder/urinary tract cancer) to 0.0% (seminoma), as shown in Figure 2.

**Figure 3:**
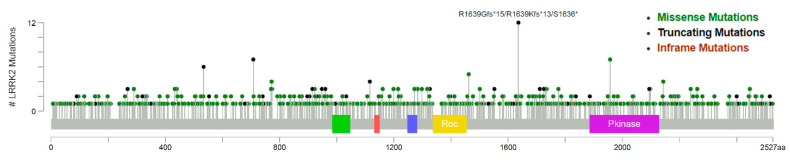
*LRRK2* missense, truncating, and inframe mutations in the studies analyzed. A scattered mutational distribution is evident. The alterations are color-coded as shown in the legend above [48,49,50].

The distribution of missense, truncating, and inframe mutations across the LRRK2 protein is shown in the lollipop plot in Figure 3. Of note, one case of endometrial carcinoma displayed the missense mutation R1441C; one case of stomach adenocarcinoma showed the missense mutation G2019D.

**Figure 4:**
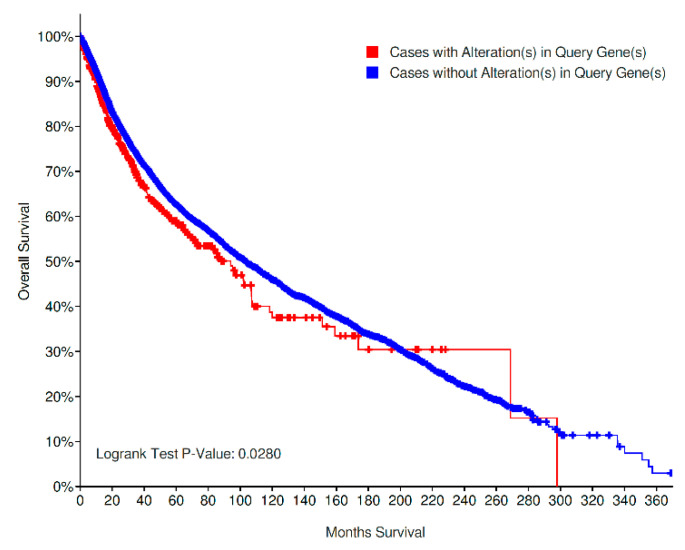
Overall survival of *LRRK2* altered (MUT and/or CNV) vs. *LRRK2* non-altered cases. A slightly worse prognosis can be observed for altered cases [48,49,50]. MUT: point mutations, indels and fusions; CNV: copy-number variations.

Overall survival (OS) analysis of all cancer types pooled together demonstrated a worse prognosis for cases with *LRRK2* alterations (207/613 deceased, median OS: 94.03 months) in comparison to cases with unaltered *LRRK2* (4123/11,356 deceased, median OS: 103.26 months) as shown in Figure 4; this result was statistically significant, with *p* = 0.0280.

**Figure 5:**
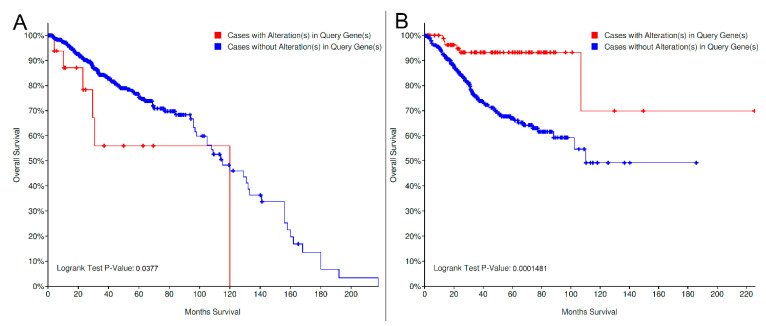
Overall survival of *LRRK2* altered (MUT and/or CNV) vs. *LRRK2* non-altered cases in prostatic adenocarcinoma (**A**) and endometrial carcinoma (**B**). The prognostic significance of *LRRK2* altered cases is negative for prostatic cancer and positive for endometrial cancer [48,49,50]. MUT: point mutations, indels and fusions; CNV: copy-number variations.

By stratifying for organ sites, a similar result was observed for prostate adenocarcinoma (*LRRK2* altered: 6/16 deceased, median OS months: 120, vs. *LRRK* non-altered: 127/649 deceased, median OS: 115.13 months; *p* = 0.037); interestingly, an opposite, statistically significant trend was noted for endometrial carcinoma (*LRRK2* altered: 6/85 deceased, median OS: NA, vs. *LRRK* non-altered: 110/479 deceased, median OS: 110.10 months; *p* = 0.0001481; Figure 5). No other significant results for other organ sites were noted, with trends pointing toward a better/worse prognosis in different settings (Figure S1).

**Figure 6:**
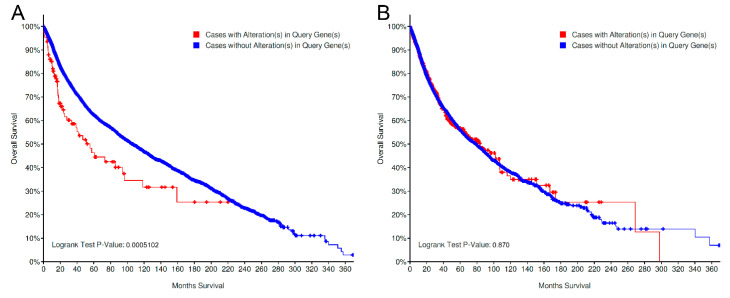
Prognostic significance of *LRRK2* CNV-altered vs. CNV-unaltered cases (**A**) and in MUT-altered vs. MUT-unaltered cases (**B**). A significant difference is noted for cases analyzed for CNVs, but not for cases profiled for MUTs [48,49,50]. MUT: point mutations, indels and fusions; CNV: copy-number variations.

When analyzing MUTs and CNVs alone, a significant (*p* = 0.0005102) worse survival was noted within the CNV-altered cases (52/111 deceased, median OS: 55.47 months) in contrast to CNV-unaltered ones (4799/13198 deceased, mean OS: 106.57 months); no difference was noted in the cohort analyzed for MUTs only (827/17454; mutated cases: 210/597 deceased, mean OS: 84 months; non-mutated cases: 3711/10945, median OS: 78.8 months; *p* = 0.870; Figure 6). The majority of CNV-altered cases included in the survival analysis are composed of *LRRK2* amplifications (85 amplifications and 26 deep deletions).

**Figure 7:**
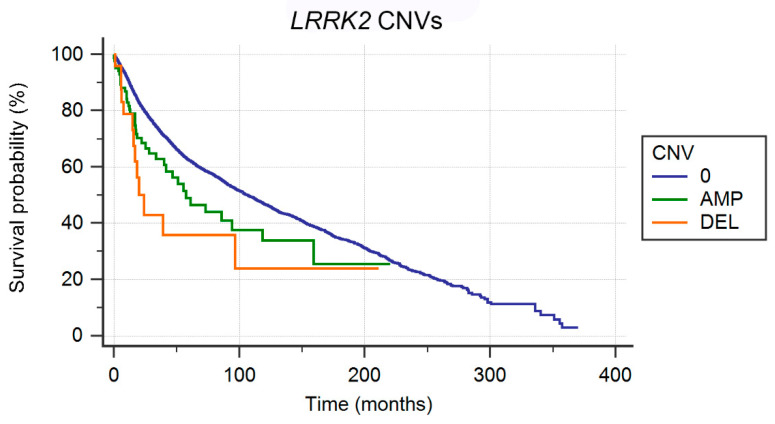
Survival of cases showing *LRRK2* whole gene deletions (DEL, orange curve), amplifications (AMP, green curve), and no copy-number alterations (0, blue curve).

By stratifying for amplifications and deletions, survival analysis shows a significantly (*p* = 0.0008) worse prognosis in terms of OS for whole gene deleted cases (13/26 deceased, median OS: 20.09 months) in comparison to amplified cases (39/85 deceased, median OS: 57.40 months) and unaltered cases (4799/13,198 deceased, median OS: 106.57 months; Figure 7).

The co-occurrence of CNV alterations of *LRRK2* and other genes located at 12q12 is shown in Table 1. The co-occurrence of CNV-alterations of *LRRK2* and other genes involved in cancer pathogenesis located at 12q13-12q15 is shown in Table 2. 

No significant clustering of *LRRK2* CNV-altered versus *LRRK2* CNV-unaltered cases was observed in terms of neoplasm staging (Figure S2). Sex and age at diagnosis distribution for *LRRK2* CNV-altered and *LRRK2* CNV-unaltered cases are shown in Figure S3; both results were statistically non-significant (*p* > 0.05).

## 4. Discussion

To our knowledge, this is the first study to assess the prevalence, characteristics, and prognostic implications of *LRRK2* alterations in a large cohort of human malignancies. A significant proportion of cancers (5%, Figure 1) across the majority of tumor subtypes (Figure 2) harbor MUTs and/or CNVs of this gene involved in the pathogenesis of PD; however, the role of *LRRK2* in cancer development remains to be elucidated, and the possible driver or passenger role of *LRRK2* alterations in cancer needs yet to be established.

The mutational landscape of *LRRK2* in this cohort suggests a passenger role of MUTs, given their scattered distribution and absence of clustering in specific domains such as Pkinase and Roc (Figure 3). It is also worth noting that the two most commonly encountered mutations of *LRRK2* in PD (and therefore pathogenic, albeit in a different setting of human disease), i.e., G2019S and R1441C, are seldom encountered (R1441C: n = 1, G2019S: n = 0, with a case showing a somatic mutation at the same codon: G2019D). An explanation could be represented by a hypothetical tumor-suppressor role of *LRRK2* in normal cellular development, for which a multitude of different inactivating mutations could result in a decrease of LRRK2 levels and activity; however, this hypothesis is not supported by previously published data [33] and our subsequent analysis.

The negative prognostic implications of *LRRK2* alterations in the studies analyzed (Figure 4) seem to suggest, on the other hand, that LRRK2 does not simply represent a bystander during the process of cancer pathogenesis; the conflicting results for prostate adenocarcinoma and endometrial carcinoma also seem to indicate a site-specific biological meaning of such alterations. By querying for MUTs and CNVs alone, the two different Kaplan–Meier plots unravel the negative prognostic role of CNV-altered cases, while the curves for mutated/nonmutated cases almost completely overlap.

By stratifying for the type of copy-number alteration, in comparison to unaltered cases, both whole gene deleted cases (CNV = −2) and amplified cases (CNV = 2) show a worse prognosis, the former being more marked. Notably, *LRRK2* amplification has been observed to be of indirect oncogenic potential in papillary renal cell carcinoma and thyroid carcinomas trough MET signaling [39].

Given the nature of the alterations which confer a negative prognostic value to *LRRK2*, i.e., amplifications and deletions, other genes located within or near the locus 12q12 need to be taken into account when trying to explain a putative role of *LRRK2* in cancer development, since duplications and deletions can involve large segments of DNA strands. The list of genes located at 12q12 and their frequency of CNV alterations in *LRRK2* CNV-altered and CNV unaltered cases is shown in Table 1; notably, all genes are significantly co-altered along with *LRRK2* (percentage of co-alterations range: 5.81–57.56%; percentage of alterations in *LRRK2* unaltered cases range: 0.07-0.54%; Log ratio range: 5.21–9.56). By querying the OncoKB database [51,52] for genes located at 12q12, only *ARID2* deletion was found to be likely oncogenic, specifically in non-small cell lung cancer [53] and hepatocellular carcinoma [54]; in our analysis, *ARID2* whole gene deletion co-occurred in 14.53% of *LRRK2* CNV-altered cases.

CNVs of other genes located at 12q12-12q15 are known to be oncogenic, such as *KMT2D* deletions [55], *ERRB3* amplifications [56], *CDK4* amplifications [57], and *MDM2* amplifications [58]. Correlations between CNV of those genes and *LRRK2* in our analysis are presented in Table 2. Notably, amplifications of *MDM2* and *CDK4* are both associated with liposarcoma [57,58]. Given the oncogenic role of amplification of *ERRB3*, *CDK4*, and *MDM2*, it could be speculated that *LRRK2* represents only a passenger of 12q amplification; it must be noted, though, that the major impact on prognosis is observed in *LRRK2* whole gene deleted cases, and the concordance between *LRRK2* and *KMT2D* whole gene deletions is 4.07% in our analysis, and a significant proportion of *LRRK2*-amplified cases lack amplification of *MDM2*, *CDK4,* and *ERBB3*. Besides, MDM2 acts as a down-regulator of p53 by initiating its ubiquitination which leads to proteasomal degradation [59], and LRRK2 has been described to phosphorylate p53 [36], therefore protecting it from MDM2-induced degradation; in this view, the 20.93% of cases harboring both *LRRK2* and *MDM2* amplifications could not rely on the latter as a driver of tumor progression. The same applies to the 15.12% of *CKD4*-amplified cases, as LRRK2 induces p21, a known CKD4 inhibitor [60], via p53 phosphorylation [36].

The hypothesized role of an increase in kinase activity of LRRK2 in cancer development, if confirmed via in vitro studies, potentially paves the way for the use of small molecule LRRK2 inhibitors beyond PD: *LRRK2*-amplificated cases are putative candidates for such treatment. Our analysis indeed shows that *LRRK2* amplification, given its negative prognostic significance, could play a role in cancer development and may be clinically actionable. And yet, we also note a dismal prognosis for *LRRK2* whole gene deleted cases. In this view, an inhibition of LRRK2 activity could prove to be more harmful than beneficial. However, when considering that both amplifications (gain of function) and whole gene deletions (loss of function) of *LRRK2* are associated with a worse prognosis in cancer, it must be acknowledged that the modulation of the kinase activity by LRRK2 small molecule inhibitors in G2019S-mutated PD patients might lead to a normal kinase function rather than a loss of kinase activity. However, given the complex interplay between the different domains of LRRK2, an inhibition of its kinase domain could have pleiotropic effects on the function of the whole protein [61,62]. Further research is needed, both in vitro and in vivo, in order to better characterize the potential anticancer effects of LRRK2 small molecule inhibitors.

Our analysis provides insights on the potential biological balancing role of *LRRK2*, a fact hinted at by the different biological processes in which this gene is involved. A number of other genes have been described to possess both oncogenic and tumor-suppressor functions [63]. A subset of these genes possesses kinase activity (e.g., *MAP2K4, MAP3K4, PRKAR1A, PRKCB*), similarly to *LRRK2*. In this view, any CNV alteration could play a role in the disruption of normal cellular homeostasis which ultimately leads to cancer. Notably, a small molecule LRRK2 inhibitor, LRRK2-IN-1, demonstrated activity in colorectal and pancreatic cancer via direct inhibition of DCLK1 [64]; the clinical utility of such therapeutic agents could also spawn further from LRRK2-overexpressed cases.

## 5. Conclusions

Herein, we demonstrated that a significant subset of human malignancies shows *LRRK2* genetic alterations. The most frequently encountered mutations in PD, G2019S, and R1441C are almost nonexistent in sporadic human malignancies. *LRRK2* CNV, both amplifications and whole gene deletions, confer a poorer prognosis in terms of OS in comparison to unaltered cases; the latter cases show the worse prognosis. In contrast, *LRRK2* somatic MUTs show no prognostic significance. These data support the fact that both LRRK2 overexpression and complete loss-of-function could play a role in cancer development, and potentially pave the way for future research to investigate the potential treatment of amplified cases with LRRK2 small molecule inhibitors; the dismal prognosis of *LRRK2* whole gene deleted cases also needs further research. In contrast, *LRRK2* non-activating MUTs are of probable passenger significance.

## Figures and Tables

**Table 1:** Co-occurrence of amplifications and whole gene deletions of genes located at 12q12 and *LRRK2*.

Gene	Alteration	Altered Group	Unaltered Group	Log Ratio	*p*-Value	*q*-Value	Enriched in
*SLC2A13*	Amp	99 (57.56%)	26 (0.17%)	8.40	5.76 × 10^−183^	2.83 × 10^−178^	Altered group
*CNTN1*	Amp	95 (55.23%)	32 (0.21%)	8.04	2.10 × 10^−170^	5.16 × 10^−166^	Altered group
*C12ORF40*	Amp	88 (51.16%)	27 (0.18%)	8.18	2.46 × 10^−158^	4.02 × 10^−154^	Altered group
*ABCD2*	Amp	85 (49.42%)	30 (0.20%)	7.98	3.71 × 10^−150^	4.55 × 10^−146^	Altered group
*KIF21A*	Amp	84 (48.84%)	35 (0.23%)	7.74	2.61 × 10^−145^	2.56 × 10^−141^	Altered group
*PDZRN4*	Amp	82 (47.67%)	35 (0.23%)	7.70	3.80 × 10^−141^	3.11 × 10^−137^	Altered group
*MUC19*	Amp	74 (43.02%)	21 (0.14%)	8.29	1.52 × 10^−132^	1.06 × 10^−128^	Altered group
*CPNE8*	Amp	82 (47.67%)	132 (0.86%)	5.79	1.09 × 10^−110^	6.65 × 10^−107^	Altered group
*ALG10B*	Amp	76 (44.19%)	137 (0.90%)	5.62	5.19 × 10^−99^	2.83 × 10^−95^	Altered group
*PPHLN1*	Amp	63 (36.63%)	54 (0.35%)	6.70	1.25 × 10^−95^	6.15 × 10^−92^	Altered group
*YAF2*	Amp	62 (36.05%)	50 (0.33%)	6.78	4.27 × 10^−95^	1.91 × 10^−91^	Altered group
*GXYLT1*	Amp	60 (34.88%)	45 (0.29%)	6.89	4.42 × 10^−93^	1.81 × 10^−89^	Altered group
*ZCRB1*	Amp	60 (34.88%)	46 (0.30%)	6.86	1.01 × 10^−92^	3.81 × 10^−89^	Altered group
*PRICKLE1*	Amp	61 (35.47%)	53 (0.35%)	6.68	3.28 × 10^−92^	1.15 × 10^−88^	Altered group
*ADAMTS20*	Amp	59 (34.30%)	61 (0.40%)	6.42	4.67 × 10^−86^	1.53 × 10^−82^	Altered group
*PUS7L*	Amp	55 (31.98%)	52 (0.34%)	6.55	1.96 × 10^−81^	6.00 × 10^−78^	Altered group
*IRAK4*	Amp	54 (31.40%)	53 (0.35%)	6.50	2.62 × 10^−79^	7.15 × 10^−76^	Altered group
*TWF1*	Amp	53 (30.81%)	57 (0.37%)	6.37	2.42 × 10^−76^	5.93 × 10^−73^	Altered group
*TMEM117*	Amp	54 (31.40%)	82 (0.54%)	5.87	5.64 × 10^−72^	1.32 × 10^−68^	Altered group
*NELL2*	Amp	53 (30.81%)	80 (0.52%)	5.88	1.08 × 10^−70^	2.40 × 10^−67^	Altered group
*CNTN1*	DeepDel	38 (22.09%)	15 (0.10%)	7.81	4.09 × 10^−64^	8.73 × 10^−61^	Altered group
*DBX2*	Amp	44 (25.58%)	62 (0.41%)	5.98	2.37 × 10^−59^	4.85 × 10^−56^	Altered group
*C12ORF40*	DeepDel	31 (18.02%)	5 (0.03%)	9.11	5.82 × 10^−57^	1.14 × 10^−53^	Altered group
*ANO6*	Amp	44 (25.58%)	76 (0.50%)	5.68	2.06 × 10^−56^	3.89 × 10^−53^	Altered group
*SLC2A13*	DeepDel	35 (20.35%)	20 (0.13%)	7.28	4.60 × 10^−56^	8.35 × 10^−53^	Altered group
*KIF21A*	DeepDel	30 (17.44%)	6 (0.04%)	8.79	3.24 × 10^−54^	5.67 × 10^−51^	Altered group
*PLEKHA8P1*	Amp	41 (23.84%)	66 (0.43%)	5.79	1.86 × 10^−53^	3.14 × 10^−50^	Altered group
*ABCD2*	DeepDel	29 (16.86%)	5 (0.03%)	9.01	5.03 × 10^−53^	8.23 × 10^−50^	Altered group
*PDZRN4*	DeepDel	32 (18.60%)	19 (0.12%)	7.22	6.05 × 10^−51^	9.57 × 10^−48^	Altered group
*SCAF11*	Amp	38 (22.09%)	77 (0.50%)	5.45	1.49 × 10^−46^	2.08 × 10^−43^	Altered group
*ADAMTS20*	DeepDel	31 (18.02%)	26 (0.17%)	6.73	1.57 × 10^−46^	2.13 × 10^−43^	Altered group
*GXYLT1*	DeepDel	28 (16.28%)	13 (0.09%)	7.58	3.17 × 10^−46^	4.15 × 10^−43^	Altered group
*ARID2*	Amp	38 (22.09%)	79 (0.52%)	5.42	3.22 × 10^−46^	4.15 × 10^−43^	Altered group
*CPNE8*	DeepDel	27 (15.70%)	10 (0.07%)	7.91	6.85 × 10^−46^	8.62 × 10^−43^	Altered group
*ALG10B*	DeepDel	25 (14.53%)	10 (0.07%)	7.79	4.00 × 10^−42^	4.90 × 10^−39^	Altered group
*LINC02402*	Amp	29 (16.86%)	31 (0.20%)	6.38	1.64 × 10^−41^	1.96 × 10^−38^	Altered group
*YAF2*	DeepDel	26 (15.12%)	16 (0.10%)	7.17	3.27 × 10^−41^	3.82 × 10^−38^	Altered group
*ZCRB1*	DeepDel	25 (14.53%)	12 (0.08%)	7.53	3.96 × 10^−41^	4.52 × 10^−38^	Altered group
*PPHLN1*	DeepDel	25 (14.53%)	14 (0.09%)	7.31	3.17 × 10^−40^	3.53 × 10^−37^	Altered group
*PRICKLE1*	DeepDel	26 (15.12%)	19 (0.12%)	6.93	4.66 × 10^−40^	5.08 × 10^−37^	Altered group
*PUS7L*	DeepDel	26 (15.12%)	22 (0.14%)	6.71	5.09 × 10^−39^	5.43 × 10^−36^	Altered group
*IRAK4*	DeepDel	25 (14.53%)	22 (0.14%)	6.66	2.89 × 10^−37^	2.78 × 10^−34^	Altered group
*NELL2*	DeepDel	24 (13.95%)	25 (0.16%)	6.41	1.25 × 10^−34^	9.57 × 10^−32^	Altered group
*RACGAP1P*	Amp	27 (15.70%)	47 (0.31%)	5.67	1.65 × 10^−34^	1.24 × 10^−31^	Altered group
*TWF1*	DeepDel	22 (12.79%)	21 (0.14%)	6.54	2.29 × 10^−32^	1.29 × 10^−29^	Altered group
*MUC19*	DeepDel	17 (9.88%)	2 (0.01%)	9.56	4.64 × 10^−32^	2.50 × 10^−29^	Altered group
*SCAF11*	DeepDel	24 (13.95%)	36 (0.24%)	5.89	6.43 × 10^−32^	3.43 × 10^−29^	Altered group
*TMEM117*	DeepDel	23 (13.37%)	30 (0.20%)	6.09	1.21 × 10^−31^	6.27 × 10^−29^	Altered group
*LINC00938*	Amp	27 (15.70%)	65 (0.43%)	5.21	1.65 × 10^−31^	8.34 × 10^−29^	Altered group
*DBX2*	DeepDel	21 (12.21%)	21 (0.14%)	6.47	1.20 × 10^−30^	5.75 × 10^−28^	Altered group
*ARID2*	DeepDel	25 (14.53%)	54 (0.35%)	5.36	3.63 × 10^−30^	1.65 × 10^−27^	Altered group
*PLEKHA8P1*	DeepDel	21 (12.21%)	25 (0.16%)	6.22	1.49 × 10^−29^	6.63 × 10^−27^	Altered group
*ANO6*	DeepDel	21 (12.21%)	27 (0.18%)	6.11	4.69 × 10^−29^	2.00 × 10^−26^	Altered group
*LINC00938*	DeepDel	21 (12.21%)	27 (0.18%)	6.11	4.69 × 10^−29^	2.00 × 10^−26^	Altered group
*RACGAP1P*	DeepDel	20 (11.63%)	21 (0.14%)	6.40	6.07 × 10^−29^	2.57 × 10^−26^	Altered group
*LINC02402*	DeepDel	10 (5.81%)	7 (0.05%)	6.99	4.09 × 10^−16^	3.74 × 10^−14^	Altered group

**Table 2:** Genes located in 12q12-12q15 for which CNVs are associated with cancer, and correlation with *LRRK2* CNVs.

Gene	Cytoband	Alteration	Altered Group	Unaltered Group	Log Ratio	*p*-Value	*q*-Value	Enriched in
*MDM2*	12q15	Amp	36 (20.93%)	480 (3.14%)	2.74	5.05 × 10^−19^	7.37 x 10−^17^	Altered group
*MDM2*	12q15	DeepDel	1 (0.58%)	4 (0.03%)	4.47	0.0544	0.0963	Altered group
*CDK4*	12q14.1	Amp	26 (15.12%)	350 (2.29%)	2.72	8.28 × 10^−14^	5.38 × 10^−12^	Altered group
*ERBB3*	12q13.2	Amp	22 (12.79%)	133 (0.87%)	3.88	2.18 × 10^−18^	2.81 × 10^−16^	Altered group
*ERBB3*	12q13.2	DeepDel	9 (5.23%)	14 (0.09%)	5.84	1.52 × 10^−12^	7.93 × 10^−11^	Altered group
*KMT2D*	12q13.12	Amp	11 (6.40%)	38 (0.25%)	4.68	4.77 × 10^−12^	2.32 × 10^−10^	Altered group
*KMT2D*	12q13.12	DeepDel	7 (4.07%)	21 (0.14%)	4.89	1.82 × 10^−8^	3.28 × 10^−7^	Altered group

## Supplementary Materials

The following are available online at https://www.mdpi.com/2073-4425/11/8/846/s1, Table S1. Studies included in the analysis using cBioPortal. Figure S1. Kaplan–Meier survival estimates of *LRRK2*-altered vs. *LRRK2*-wild-type cases in (A) urothelial cancer, (B) esophagogastric cancer, (C) melanoma, (D) non-small cell lung cancer, (E) colorectal cancer, and (F) pancreatic cancer [48,49,50]. Figure S2. Neoplasm staging for *LRRK2* CNV-altered and *LRRK2* CNV-unaltered cases. No significant clustering can be observed [48,49,50]. Figure S3. Sex (A) and age at diagnosis (B) distribution for LRRK2 CNV-altered and LRRK2 CNV-unaltered cases (A, *p* > 0.05, Chi-squared test; B, altered group: median 61.2, interquartile range 52.1-65.0; unaltered group, median 61.16, interquartile range 52.0-69.6; *p* > 0.05, Kruskal–Wallis Test) [48,49,50].
