# Supplementary figures and images for: Comprehensive Genomic Analysis Reveals the Prognostic Role of LRRK2 Copy-Number Variations in Human Malignancies
Source: Genes (Basel). 2020 Jul 24;11(8):846. doi: 10.3390/genes11080846 (PMC7465025; doi:10.3390/genes11080846)

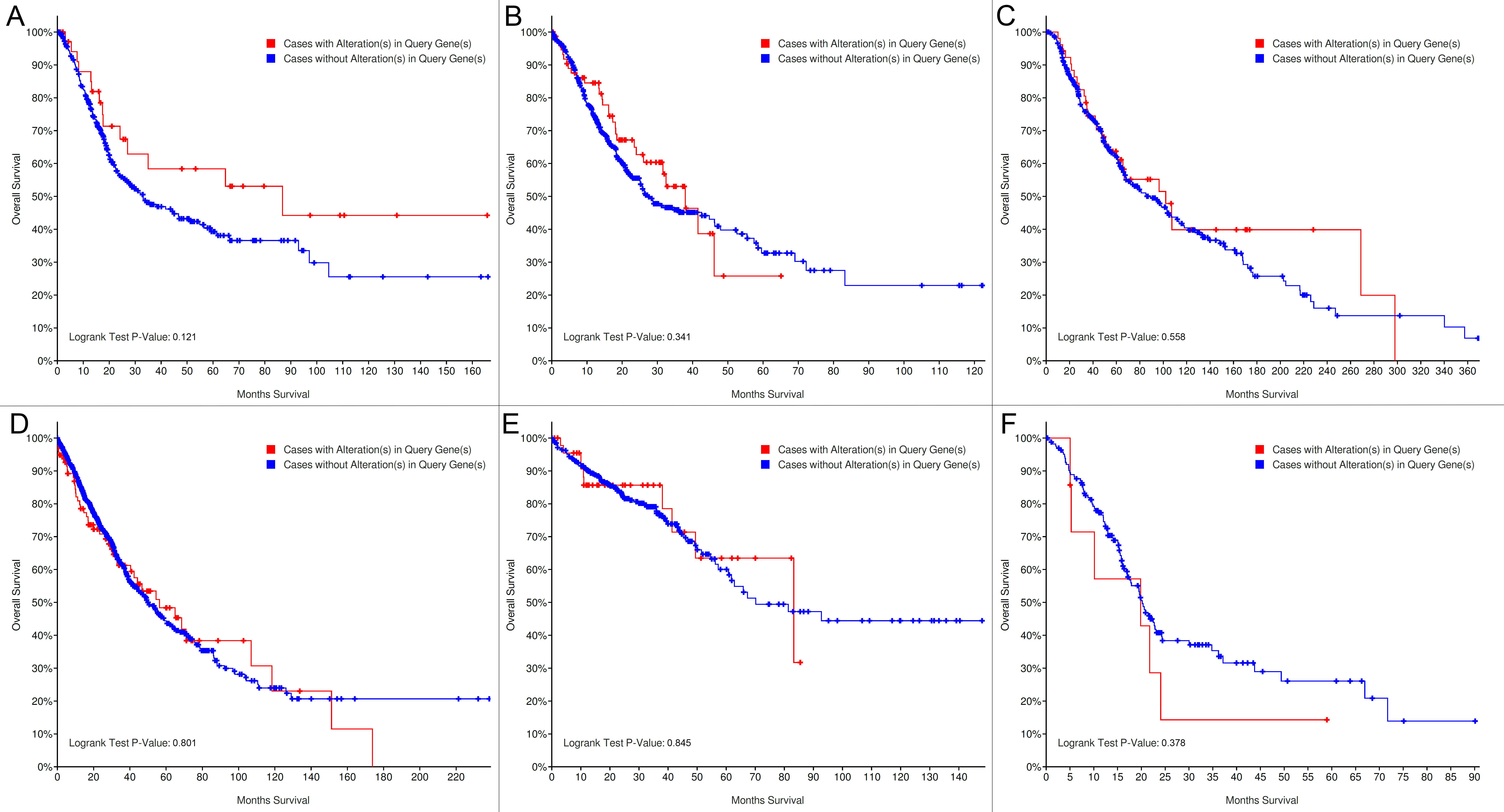

Supplement: Supplementary file 1 [file genes-11-00846-s001.zip › Supplementary Figure 1.tif]

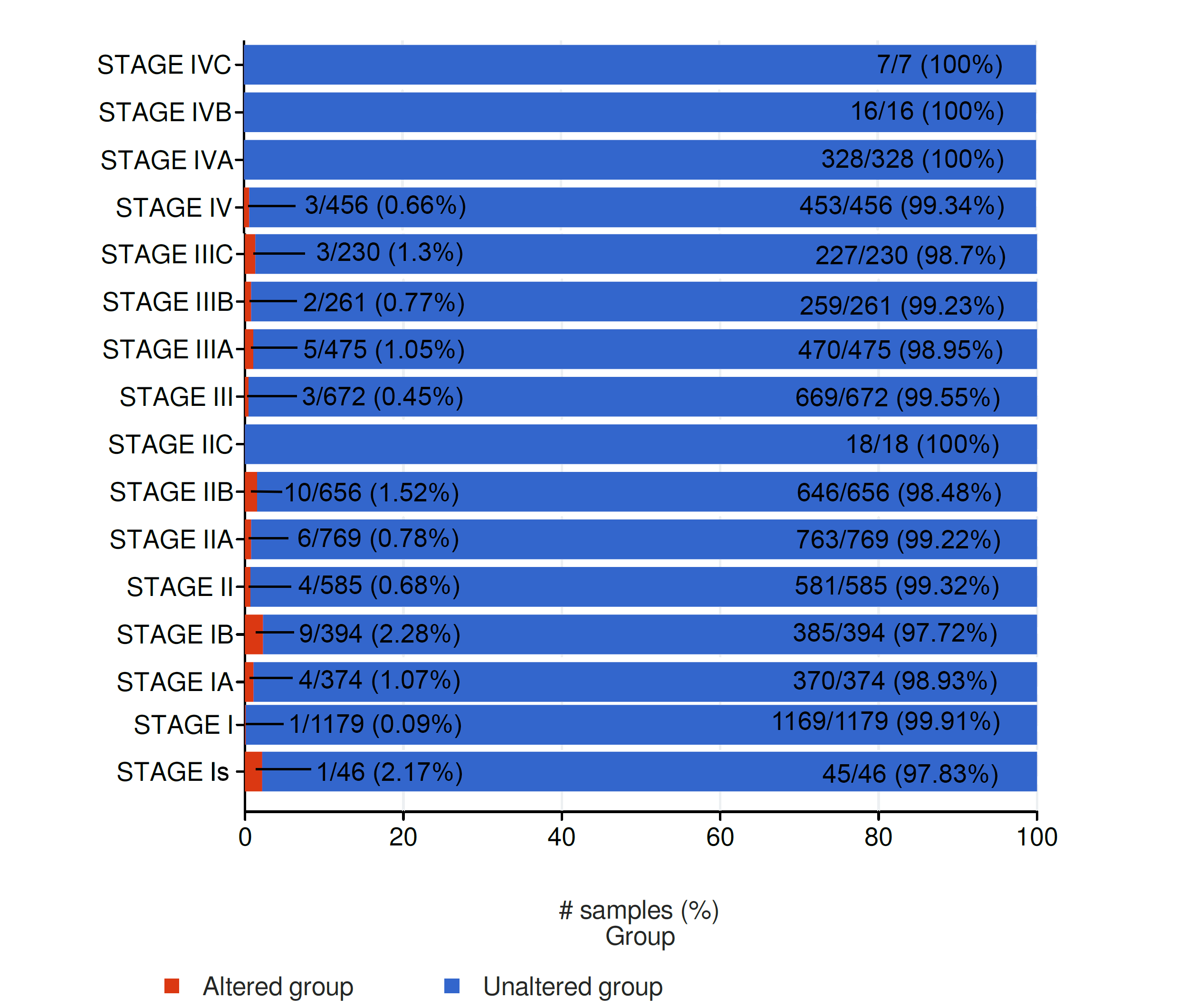

Supplement: Supplementary file 1 [file genes-11-00846-s001.zip › Supplementary Figure 2.tif]

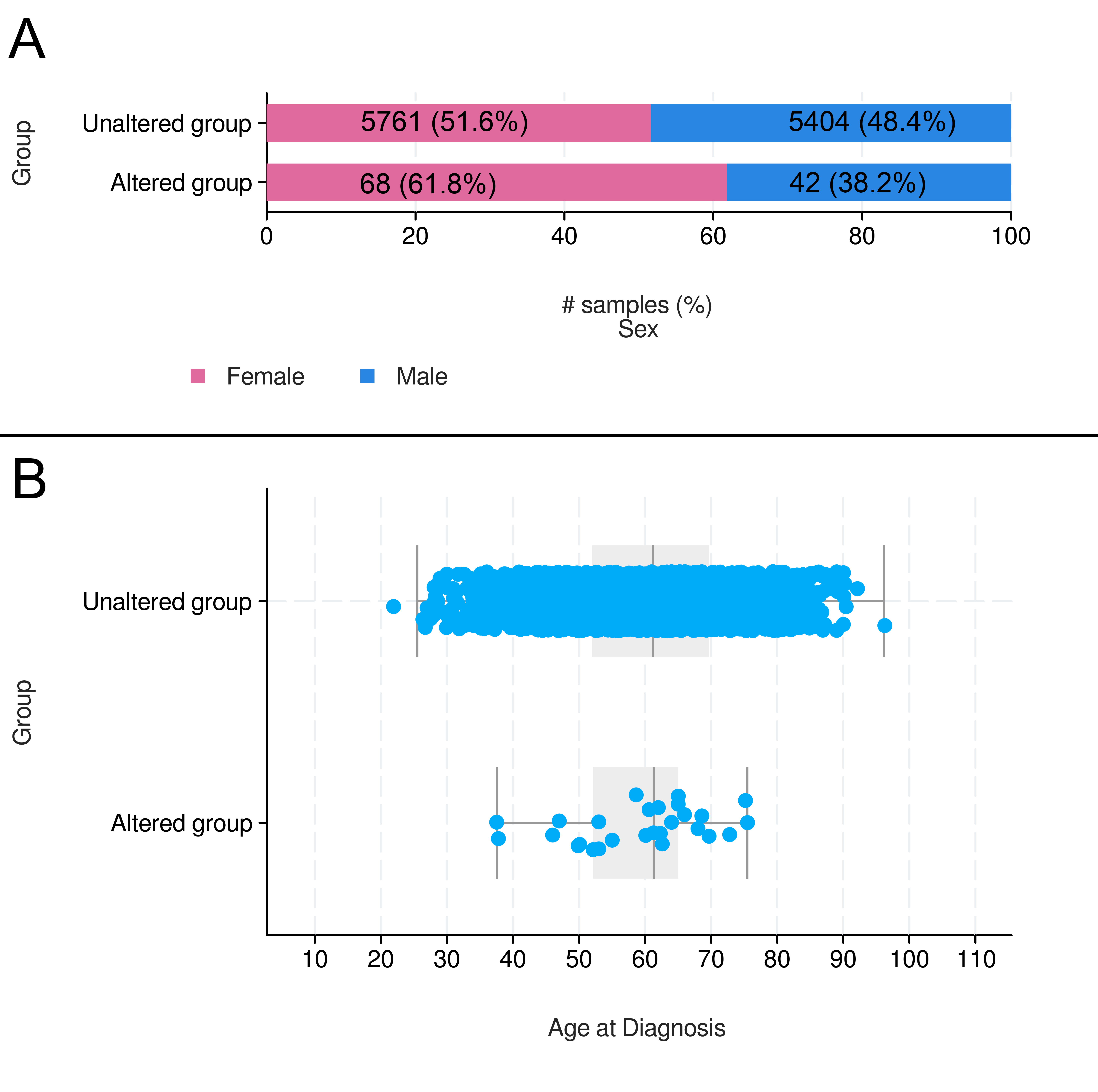

Supplement: Supplementary file 1 [file genes-11-00846-s001.zip › Supplementary Figure 3.tif]
